# Effects of white-tailed deer habitat use preferences on southern cattle fever tick eradication: simulating impact on “pasture vacation” strategies

**DOI:** 10.1186/s13071-021-04590-z

**Published:** 2021-02-08

**Authors:** M. Sofia Agudelo, William E. Grant, Hsiao‑Hsuan Wang

**Affiliations:** 1 Department of Wildlife and Fisheries Sciences, Texas A&M University, College Station, TX 77843 USA; 2 Present Address: Western EcoSystems Technology, Inc. (WEST), Bismarck, ND 58503 USA; 3 Department of Ecology and Conservation Biology, Texas A&M University, College Station, TX 77843 USA

**Keywords:** Cattle Fever Tick Eradication Program, Host–parasite interaction, Individual-based model, Integrated tick management research, *Rhipicephalus* sp., Spatially explicit model

## Abstract

**Background:**

*Rhipicephalus* (*Boophilus*) *annulatus* and *Rhipicephalus* (*Boophilus*) *microplus* (southern cattle fever tick; SCFT), collectively known as cattle-fever ticks (CFTs), are vectors of protozoal parasites (*Babesia bigemina* and *Babesia bovis*) that cause bovine babesiosis (also known as cattle fever). One traditional strategy for CFT eradication involves the implementation of a “pasture vacation,” which involves removing cattle (*Bos taurus*) from an infested pasture for an extended period of time. However, vacated pastures are often inhabited by wildlife hosts, such as white-tailed deer (WTD; *Odocoileus virginianus*), which can serve as alternate hosts for questing CFTs. We hypothesized that the distribution of host-seeking larvae among habitat types post-pasture vacation would reflect habitat use patterns of WTD, and in turn, affect the subsequent rate of pasture infestation by CFT.

**Methods:**

We adapted a spatially explicit, individual-based model to simulate interactions among SCFT, cattle, and WTD as a tool to investigate the potential effects of WTD habitat use preferences on the efficacy of a pasture vacation. We parameterized the model to represent conditions typical of rangelands in south Texas, USA, simulated a 1-year pasture vacation under different assumptions regarding WTD habitat use preferences, and summarized effects on efficacy through (1) time post-vacation to reach 100% of pre-vacation densities of host-seeking larvae, and (2) the ecological conditions that resulted in the lowest host-seeking larval densities following pasture vacation.

**Results:**

Larval densities at the landscape scale varied seasonally in a similar manner over the entire simulation period, regardless of WTD habitat use preferences. Following the removal of cattle, larval densities declined sharply to < 100 larvae/ha. Following the return of cattle, larval densities increased to > 60% of pre-vacation densities ≈ 21 weeks post-vacation, and reached pre-vacation levels in less than a year. Trends in larval densities in different habitat types paralleled those at the landscape scale over the entire simulation period, but differed quantitatively from one another during the pasture vacation. Relative larval densities (highest to lowest) shifted from (1) wood/shrub, (2) grass, (3) mixed-brush during the pre-vacation period to (1) mixed-brush, (2) wood/shrub, (3) grass or (1) wood/shrub, (2) mixed-brush, (3) grass during the post-vacation period, depending on WTD habitat use preferences.

**Conclusions:**

By monitoring WTD-driven shifts in distributions of SCFT host-seeking larvae among habitat types during simulated pasture vacation experiments, we were able to identify potential SCFT refugia from which recrudescence of infestations could originate. Such information could inform timely applications of acaricides to specific refugia habitats immediately prior to the termination of pasture vacations.
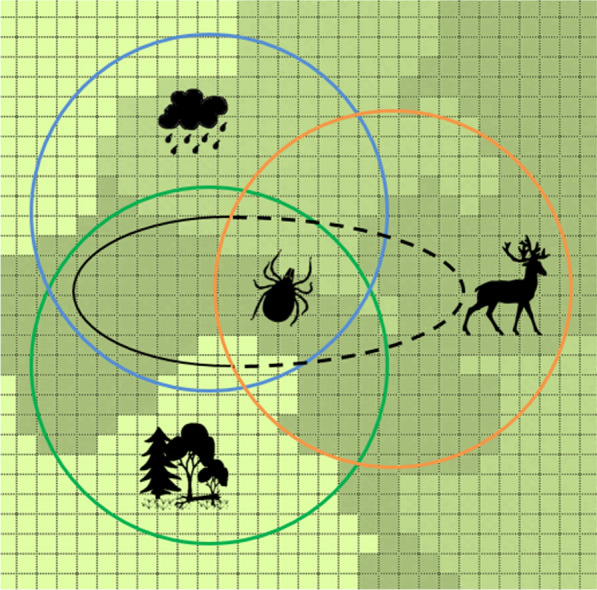

## Background

*Rhipicephalus* (*Boophilus*) *annulatus* and *Rhipicephalus* (*Boophilus*) *microplus* (southern cattle fever tick; SCFT), collectively known as cattle-fever ticks (CFTs), are vectors of protozoal parasites (*Babesia bigemina* and *Babesia bovis*) that cause bovine babesiosis (cattle fever), which is considered the most economically important livestock disease worldwide [1]. Thus, keeping cattle (*Bos taurus*) herds free of bovine babesiosis is an important economic and animal health priority [2, 3]. CFT and *Babesia* sp. are prevalent in Mexico, but since the 1940s have been confined in the USA primarily within the permanent CFT quarantine zone, which is along the border with Mexico and is maintained by the Cattle Fever Tick Eradication Program (CFTEP) [4]. However, in 2009, an area outside the permanent quarantine zone covering > 400,000 hectares (ha) in south Texas, USA, was quarantined due to CFT infestations [5]; as of 2019, ≈ 300,000 ha outside the permanent quarantine zone is under CFT quarantine [6].

Historically, the success of the CFTEP has depended largely on the host specificity of CFTs [7]. CFTs are one-host ticks that complete their life cycle on a single host. Off-host (host-seeking) larvae attach to a host, feed and molt to nymphs (1 week), and then feed again and molt to adults (1 week) on the same host animal. Adult females mate and engorge on the host (1–2 weeks), then detach and fall to the ground where they lay large egg masses [8] and die, usually within a few days. Survival of off-host life stages (eggs, larvae) is affected by the climatic conditions to which they are exposed, which varies depending on the habitat type in which the eggs were laid. Further, the location of egg masses reflects the habitat use of hosts, and the likelihood of CFTs encountering host species depends on host community composition and density [9, 10].

Climate variables such as temperature, saturation deficit, and precipitation are known to affect the survival and development of CFT [11, 12]. Although publicly available information on host-seeking larvae densities does not exist for south Texas, simulation modeling approaches under typical weather patterns of south Texas have shown that CFT populations oscillate in response to favorable conditions for host-seeking larval survival in spring and fall (characterized by increased precipitation and low saturation deficit), and unfavorable conditions in summer and winter (characterized by high and low temperatures compounded with varying conditions of precipitation and saturation deficit) [10]. Studies have suggested that the interaction between weather and habitat variables strongly influences the survival of host-seeking larvae in south Texas [13]. Information provided by Teel et al. [9, 14, 15] indicates that canopy cover attributes of different habitat types interact with abiotic factors to characterize the landscape as good, fair, or poor habitats for CFT survival and development. For example, habitat suitability in south Texas rangelands varies from good in wood/shrub-canopied habitats, to fair in mixed-brush habitats, to poor in uncanopied grass habitats.

Although cattle are the main CFT hosts, the white-tailed deer (WTD; *Odocoileus virginianus*) is also a confirmed host [16]. Serologic and molecular evidence suggests that WTD in northern Mexico and southern Texas carry bovine babesiosis [17], and genetic data suggest WTD likely serve as a source for tick populations that will eventually feed on cattle [18]. Both field and modeling studies have examined the potential role of ungulate hosts related to disease transmission [19], the effects of seasonal fluctuations in host communities on the dynamics of infectious disease [20], and the efficacy of targeting acaricides at specific types of hosts [21]. Wang et al. [10] focused specifically on modeling the impact of interactions between cattle and WTD on SCFT-eradication methods. These authors suggested that WTD could reduce the efficacy of eradication efforts by maintaining viable tick populations during eradication treatments aimed at cattle [10].

Two traditional methods for CFT eradication involve the use of acaricides and implementation of a “pasture vacation” [22], with the latter defined as the removal of cattle from a pasture for an extended length of time [23, 24]. The efficacy of acaricides is potentially compromised by several factors, including the evolution of resistance of CFTs to them [25], a limited ability to apply acaricides to wildlife hosts [26], and the existence of diverse plant communities that provide an abundance of habitats favorable for the survival of CFTs [3]. Acaricide methods employed by the CFTEP to treat cattle on infested premises require treatment of the entire herd at 2-week intervals to assure 100% CFT elimination [27]. The practice of vacating pastures also remains a viable option in the CFTEP, although there have been an increasing number of failures of this method, which often have been related to the presence of WTD [16].

Epidemiological analysis of CFT infestations have indicated that WTD can compromise eradication efforts by sustaining and dispersing CFTs within and outside the permanent quarantine zone [16]. The degree to which WTD can sustain or re-infest an area with CFTs depends in large part on the distribution of different habitat types across the landscape. Habitat use preferences of WTD, although widely studied [28–31], are poorly understood within the context of CFT management strategies. Field studies report habitat use patterns with varying degrees of specificity, which are case specific and difficult to compare due to the inevitable confounding effect of habitat preference versus availability. Nonetheless, some general patterns emerge. Pollock et al. [32] reported that in south Texas the areas more heavily used year-round by WTD were characterized by shrub vegetation with high canopy cover and high woody species diversity. Avery et al. [33] found that WTD in west-central Texas spent 54% of their time in woody (shrub) areas. DeYoung et al. [34] reported that WTD in south Texas spent 65–76% of their time in woody (shrub) areas depending on their own nutritional status.

The importance of the explicit consideration of habitat use by alternative hosts such as WTD when planning efforts to eliminate CFT infestations has been become a topic of much interest [3]. Wang et al. [10] suggested that WTD could undermine the efficacy of CFT eradication efforts by dispersing engorged female ticks into, and collecting host-seeking larvae from, habitats favorable for the survival and development of off-host life stages, thus creating tick refugia during eradication treatments aimed at cattle. In south Texas rangelands, WTD use habitats characterized by shrub vegetation with high canopy cover and high woody species diversity [32, 34]. These canopied plant communities provide habitats favorable for the survival and development of off-host CFT life stages, and also provide good browse for WTD (all of the shrubs mentioned by these authors in these plant communities are palatable to WTD except for whitebrush *Aloysia gratissima*). Thus, exploration of the WTD-mediated distribution of host-seeking CFT larvae during and immediately following pasture vacation has the potential to suggest novel approaches to the elimination of CFT infestations.

In the present study, we adapted the model of Wang et al. [10] to investigate potential effects of WTD habitat preferences on the eradication of SCFT infestations. More specifically, we simulated the effects of changes in WTD habitat preferences for good SCFT habitat (wood/shrub) on the distribution of host-seeking larvae during and after a 1-year pasture vacation, applied under climate and landscape conditions typical of rangelands in south Texas.

## Methods

### Model description

**Fig. 1:**
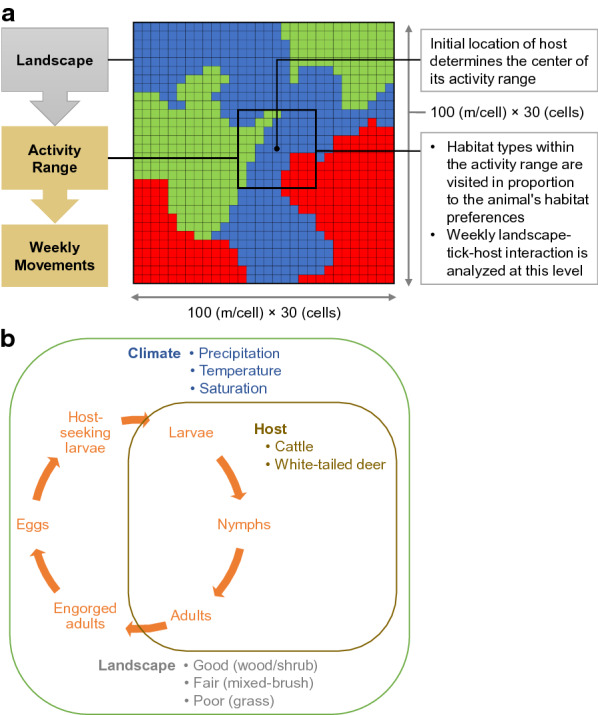
**a** Schematic representation of the hierarchical relationships among the 900-ha simulated landscape, host activity ranges, and weekly host movements. **b** Conceptualization of the tick-host-landscape-climate system indicating important model components and processes

**Fig. 2:**
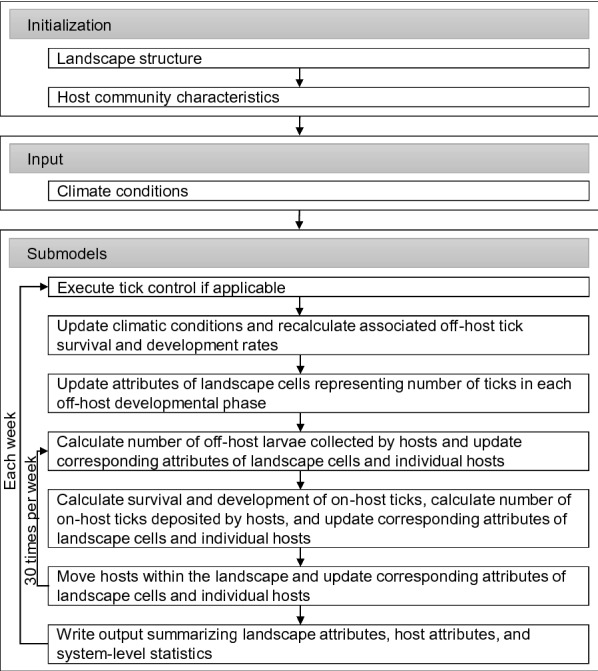
Overview of the sequence of events and processes involved in the execution of the model simulating the spatial and temporal dynamics of southern cattle fever ticks (SCFTs) (Adapted and modified from Wang et al. [10])

To investigate potential effects of WTD habitat use on SCFT-eradication efforts, we used the spatially explicit, individual-based model of Wang et al. [10]. The model is designed to simulate the effects of shifts in the spatial-temporal patterns of host (cattle and WTD) habitat use on the dynamics of SCFT populations (Fig. 1). A complete model description following the Overview, Design concepts, Details protocol [35, 36] is provided in Wang et al. [10]. Figure 2 lists the steps involved in the model’s execution. Below, we provide a summary of the detailed Overview, Design concepts, Details model description, following the format suggested by Grimm et al. [37]. The format focuses on model (1) purpose, (2) patterns, (3) entities, (4) state variables, (5) spatial and temporal scales, (6) processes, and (7) design concept. We have italicized these terms in the description below.

The overall *purpose of the model* is to examine how the presence of WTD affects the efficacy of pasture vacation as a CFT eradication strategy in south Texas. Specifically, we hypothesized that the post-vacation distribution of host-seeking larvae among habitat types resulting from habitat use patterns of WTD affects subsequent patterns of pasture infestation. We consider the model realistic enough for this purpose based on its ability to generate ecologically interpretable spatial-temporal distribution *patterns of host-seeking larvae* under rangeland conditions typical of south Texas: (1) the mean density of host-seeking larvae exhibits oscillations consistent with their exposure to environmental conditions during the off-host phase of their life cycle (Fig. 4b in Wang et al. [10]); (2) annual variation in host-seeking larvae follows a bimodal pattern, with a spring increase and summer decline, followed by a fall increase and winter decline (Fig. 4e, f in Wang et al. [10]); (3) the highest density host-seeking larvae populations occur in wood/shrub habitats and along edges of mixed-brush, which results from heavier usage of grass and mixed-brush habitats by cattle, and heavier usage along edges of mixed-brush and wood/shrub by WTD (Fig. 5 in Wang et al. [10]).

*Entities* represented in the model include (1) 900, square, 1-ha habitat cells, and (2) several hundred individual mammalian hosts (cattle and WTD). *State variables* of habitat cells include location, habitat type (wood/shrub, mixed-brush, or grass), and current numbers of SCFT eggs, larvae, and engorged (fed) adults in each weekly age-class located in the cell, as well as the current numbers of cattle and WTD located in the cell. *State variables* of individual hosts include the location of the center of activity range, current location, habitat type of current location, relative habitat preference [low (0) to high (1)] for each habitat type, size of activity range (ha), relative number of larvae they can carry at any given time (1.0 for cattle, 0.1 for WTD), and numbers of larval, nymphal, and adult SCFTs in each age-class that hosts currently carry. *Spatial scales* are defined in terms of the 900, square, 1-ha habitat cells. The *temporal scales* explored are a weekly time step for a total of 3 years for calculation of SCFT development and survival. However, hosts move about the landscape 30 times per week, acquiring and depositing SCFTs among the habitat cells they visit.

The most important *processes* represented in the model include (1) temperature-dependent and habitat-specific development and survival of off-host SCFTs, (2) host-specific survival of on-host SCFTs and host-specific fecundity of deposited (fed) SCFTs, (3) the movement of hosts based on habitat preferences, and (4) host-specific and habitat-specific acquisition and deposition of SCFTs. Tick development, survival, and reproduction occur at the beginning of each weekly time step. Host movement, tick acquisition, and tick deposition occur 30 times within each weekly time step. (Note that tick acquisition and deposition do not necessarily occur with each individual host movement).

The most important *design concept* is that the SCFT life cycle is influenced by climatic conditions, landscape structure, and the composition of the host community. Survival and development of off-host stages are dependent on the temperature and relative humidity in habitat types to which they are exposed as a consequence of host-driven dispersal of gravid females. Completion of the SCFT life cycle depends on larvae encountering an appropriate host, which is influenced by species abundance, composition of the host community, and habitat preferences of potential host species. Spatial and temporal patterns of abundance of off-host SCFT larvae emerge as system-level properties as a result of equations describing rates of off-host tick development and survival, and rules governing the movements of hosts within the landscape (Additional file 1).

### Model application

**Fig. 3:**
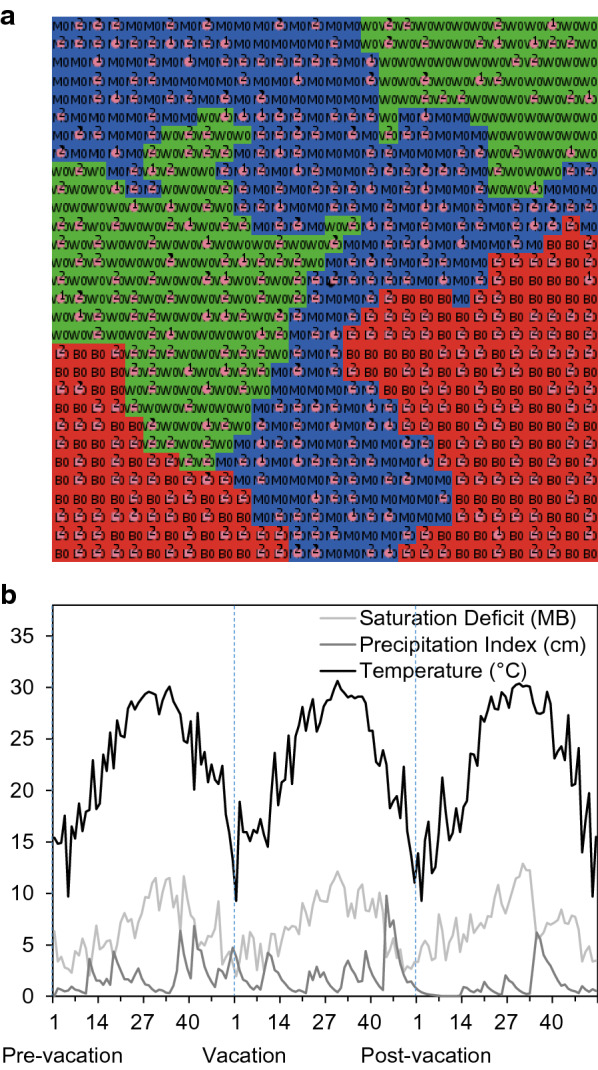
**a** Spatial distribution of habitat types across the simulated landscape, consisting of 30% wood/shrub (good SCFT) habitats (*green* with* W0*), 30% mixed-brush (fair SCFT) habitats (*red* with* B0*), and 40% grass (poor SCFT) habitats (*blue* with* M0*).* Pink dots* with the number* 1* represent cattle, *pink dots* with the number* 2* represent white-tailed deer (WTD). **b** Weather profile during the 3-year simulation period based on historical temperature, saturation deficit, and precipitation data for Corpus Christi, Texas, USA.* Vacation* “Pasture vacation”

For the present application, we characterized the modeled landscape on the basis of the dominant vegetation types (sensu McMahan [38]), which consisted of 30% wood/shrub community dominated by mesquite (wood/shrub), 30% mixed-brush community dominated by thorn shrubs (mixed-brush), and 40% meadow community dominated by grasses, forbs, and grass-like plants (grass) (Fig. 3a). Following Wang et al. [10], we assumed that wood/shrub, mixed-brush, and grass communities represented good, fair, and poor habitats, respectively, with regard to survival rates of host-seeking tick larvae. We modified the representation of WTD habitat use preference for good SCFT habitat such that it could be altered from no use to exclusive use (i.e. WTD could spend 0–100% of their time in good SCFT habitat), with the remaining use preferences split equally between fair and poor habitats. Habitat use preferences of cattle reflected values from Wang et al. [10]: 0.3 for good (wood/shrub), 0.1 for fair (mixed-brush), and 0.6 for poor (grass) SCFT habitat. When present, host densities (0.125 cattle/ha, 0.6175 WTD/ha) and activity ranges (300 ha for cattle, 675 ha for WTD) also reflected values from Wang et al. [10].

We simulated a 1-year pasture vacation experiment under different assumptions regarding WTD habitat use preferences. We used a 3-year simulation period (pre-vacation year, vacation year, and 1st year post-vacation) in which cattle were removed from the system at the beginning (1st week in January) of the vacation year, and were restocked at the beginning of the 1st year post-vacation. The experimental design consisted of 5 Monte Carlo simulations for each of 11 WTD habitat use preferences for good SCFT habitat (0–1 in increments of 0.1, e.g. when preference was 0, WTD did not enter good SCFT habitat; when preference was 0.5, WTD spent ≈ 50% of their time in good SCFT habitat; when preference was 1, WTD did not leave good SCFT habitat). As in Wang et al. [10], simulated climatic conditions were generated based on historical temperature, saturation deficit, and precipitation data for Corpus Christi, Texas (Fig. 3b). During each simulation, we monitored numbers of host-seeking larvae in each landscape cell each week. We summarized results in terms of (1) the number of weeks following the end of the pasture vacation needed to reach 100% of the pre-vacation host-seeking larval density at the landscape scale; (2) the lowest mean host-seeking larval densities at the landscape scale and in each habitat type following the end of pasture vacation; and (3) spatial distributions of host-seeking larvae from immediately pre-vacation through 1-year post-vacation.

## Results

**Fig. 4:**
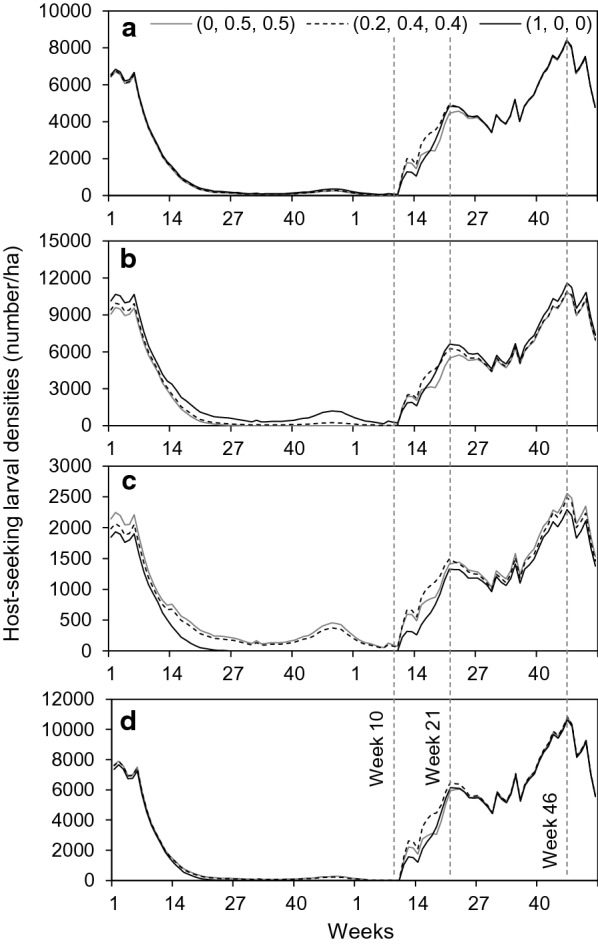
**a**–**d** Temporal response of SCFT host-seeking larval densities under different WTD habitat use preferences during the vacation and post-vacation years (pre-vacation year not shown). Weekly mean numbers of host-seeking larvae per ha are shown at the landscape scale (**a**) and by habitat type: wood/shrub (**b**), mixed-brush (**c**), and grass (**d**). Results are from simulations in which WTD habitat use preferences for wood/shrub (good SCFT) habitats, mixed-brush (fair SCFT) habitats, and grass (poor SCFT) habitats were 0, 0.5, 0.5 (*gray solid line*), 0.2, 0.4, 0.4 (*dashed line*), and 1, 0, 0 (*black solid line*), respectively.* Dashed vertical lines* representing weeks 10, 21, and 46 of the post-vacation year are included for reference

Mean weekly host-seeking larval densities at the landscape scale varied seasonally in a similar manner regardless of WTD habitat use preferences (Fig. 4a). Larval densities declined sharply within the first 6 weeks of pasture vacation, and continued decreasing to lows of < 100/ha (≈ 65–95/ha, depending on WTD preferences) by week 34. Larval densities then increased slightly during a period of increased precipitation and moderate temperatures during the last quarter of the vacation year, and finally decreased again to below 100/ha. Following the return of cattle (1st week in January of the post-vacation year) and onset of warmer temperatures (≈ 10 weeks post-vacation), larval densities began increasing from their post-vacation lows and recovered to > 60% of pre-vacation densities (≈ 4800/ha) ≈ 21 weeks post-vacation. In less than a year (≈ 46 weeks post-vacation), larval densities reached pre-vacation levels (≈ 8000/ha), and resumed typical seasonal and year-to-year fluctuations thereafter.

Trends in larval densities in different habitat types paralleled those at the landscape scale during all scenarios (Fig. 4b–d). Not surprisingly, as WTD habitat use preferences for wood/shrub increased (from 0 to 1) across scenarios, larval densities increased in wood/shrub habitats and decreased in mixed-brush and grass habitats. When preference for wood/shrub was 0 (WTD spent 50% of time in mixed-brush and 50% in grass), larval densities in wood/shrub decreased to lows of ≈ 50/ha, and to lows of ≈ 50/ha and ≈ 1/ha in mixed-brush and grass, respectively. When WTD habitat use preference for wood/shrub was 1 (WTD used wood/shrub exclusively), larval densities during the vacation year decreased to lows of ≈ 200/ha in wood/shrub, and SCFTs were eliminated from mixed-brush and grass habitats. However, following the return of the cattle and the onset of warmer temperatures, larval density differences among habitats essentially disappeared.

**Fig. 5:**
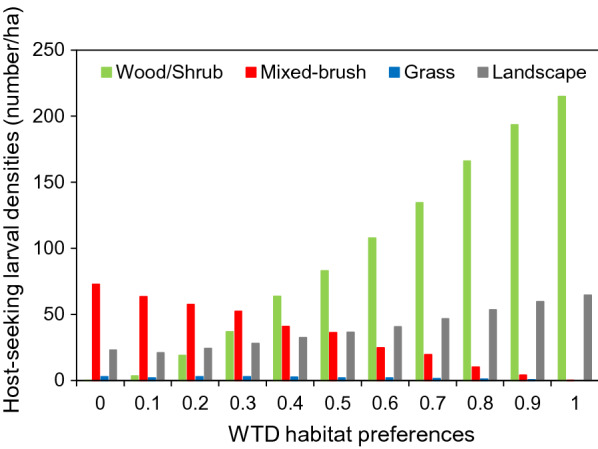
SCFT host-seeking larval densities at the landscape scale and in each habitat type at their lowest point, 10 weeks post-pasture vacation, under the indicated assumptions regarding WTD habitat use preferences. Preference values represent the proportion of time spent in wood/shrub (good SCFT) habitats, with the remaining portion of time divided equally between mixed-brush (fair SCFT) habitats and grass (poor SCFT) habitats

Distribution of larval densities among habitat types at their lowest point, 10 weeks post-vacation, was higher in mixed-brush when WTD habitat use preferences for wood/shrub were ≤ 0.3 and was higher in wood/shrub when preferences were ≥ 0.4; larval densities always were lowest in grass (Fig. 5). Thus, relative larval densities (highest to lowest) shifted from wood/shrub, grass, mixed-brush during the pre-vacation period to mixed-brush, wood/shrub, grass or wood/shrub, mixed-brush, grass during the post-vacation period, depending on WTD habitat use preferences.

**Fig. 6:**
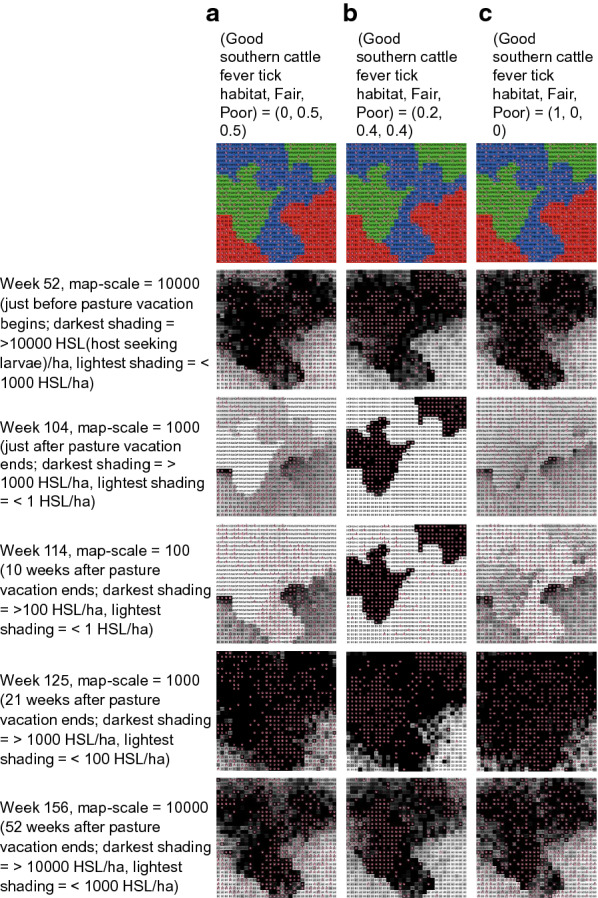
Temporal shifts in spatial distributions of SCFT host-seeking larval densities [host-seeking larvae (*HSL*)/ha] across a simulated landscape assuming different WTD habitat use preferences. The simulated landscape consisted of 30% wood/shrub (good SCFT) habitats (*green*), 30% mixed-brush (fair SCFT) habitats (*red*), and 40% grass (poor SCFT) habitats (*blue*). Results are from simulations in which WTD habitat use preferences for wood/shrub, mixed-brush, and grass were **a** 0, 0.5, 0.5, **b** 0.2, 0.4, 0.4, and **c** 1, 0, 0, respectively.* Darker shading* indicates higher densities of HSL. Note differences in shading scales

Spatial distributions of host-seeking larvae immediately pre-vacation and 1-year post-vacation were similar, regardless of WTD habitat use preferences; however, distributions immediately following the vacation period differed depending on preferences (Fig. 6). The rate of spatial expansion post-vacation of relatively densely infested areas was similar regardless of preferences, and by week 21 post-vacation, spatial distributions of host-seeking larvae also were similar.

## Discussion

The simulated effects of WTD habitat use preferences on the efficacy of pasture vacation can be summarized by shifts in the distribution of host-seeking larvae among habitat types during the pasture vacation. These shifts in larval distributions did not have the hypothesized effect on subsequent rates of pasture infestation. Rather, they provided potential SCFT refugia from which recrudescence of infestations could originate. Pre-vacation distributions of host-seeking larvae primarily reflected habitat use preferences of cattle, superimposed on habitat-specific differences in larval survival, thus masking the role of WTD in the maintenance of larvae. Larval densities were highest in wood/shrub, which was used relatively frequently (≈ 30% of the time) by cattle and in which larval survival was highest, followed by grass, which was used most frequently (≈ 60%) by cattle but in which larval survival was lowest. Larval densities were lowest in mixed-brush, where survival was intermediate and cattle use was lowest (≈ 10%). Post-vacation, before tick population recovery began, relative larval densities primarily reflected habitat use preferences of WTD, again superimposed on habitat-specific differences in larval survival. When WTD preferences were close to those assumed by Wang et al. [10] (≈ 20% of time in wood/shrub, ≈ 40% in mixed-brush, ≈ 40% in grass), larval densities were highest in mixed-brush. If time spent in wood/shrub was roughly double that assumed by Wang et al. [10], larval densities were highest in wood/shrub. If time spent in wood/shrub was lower than that assumed by Wang et al. [10], host-seeking larvae were essentially confined within mixed-brush refugia.

The potential for the existence of tick refugia is inherent in the heterogeneous distribution of favorable tick habitats and varied patterns of landscape use by cattle and WTD [39]. Landscape use by cattle and WTD in south Texas rangelands has been the subject of many investigations focused on animal production or conservation goals [29, 39–41]. Species-specific preferences for forage or browse create spatial layers of landscape use, which, in combination with spatially variable physical environmental conditions, form the tick-host-landscape mosaic where CFT populations exist [42, 43], and where CFT eradication efforts operate [3].

By monitoring the WTD-driven shifts in distributions of host-seeking larvae among habitat types during our simulated pasture vacation experiments, we were able to identify potential SCFT refugia from which recrudescence of infestations could originate following reintroduction of cattle. The importance of feedback mechanisms at the habitat–wildlife–livestock interface in the control of infectious diseases is recognized globally [44–47]. Pérez de León et al. [3] have stressed the importance of explicit consideration of habitat use patterns of wildlife hosts in the development of integrated strategies for CFT eradication. Recent studies have focused on movements of WTD as hosts of CFTs [26], while others have highlighted the ecological plasticity of CFTs [48], as factors complicating tick control measures. Within the present context, our model provides an investigative tool that could inform, for example, timely applications of acaricides to specific refugia habitats immediately prior to termination of pasture vacations [49]. Future model applications might simulate management schemes conducted on real landscapes that included different combinations and densities of potential wildlife host species. Of high priority would be simulations assessing the efficacy of acaricide applications to, and culling of, wildlife hosts on landscapes located near the permanent CFT quarantine zone, where nilgai (*Boselaphus tragocamelus*) have been implicated in potential CFT reinfestations [7]. We hasten to add that our model is not intended to make precise predictions, but, rather, to provide an exploratory tool with which to investigate potential effects of habitat preferences of WTD and other potential wildlife hosts on the efficacy of pasture vacations.

## Conclusions

Although cattle are the main hosts of CFT, WTD are also confirmed hosts in and around the permanent CFT quarantine zone along the US-Mexico border. Recent CFT infestations outside the quarantine zone pose a serious threat to the US cattle industry. The effects of interactions among host species composition, habitat heterogeneity, and climatic variability on the efficacy of CFT eradication strategies are virtually impossible to investigate in the field. The effects of habitat use preferences of WTD, as well as other potential wildlife hosts, are poorly understood within the context of CFT management. Spatially explicit, individual-based models such as the present one are useful tools for identifying CFT distribution patterns that emerge from a wide variety of tick-host-habitat-climate interactions. Such models allow preliminary a priori evaluation of alternative eradication strategies, including novel strategies involving new technologies, which might require significant investment before a field trial would be possible.

## Supplementary Information

**Additional file 1.** Summary of model parameters and equations.

## Data Availability

Not applicable.

## Abbreviations

CFT: Cattle fever tick
CFTEP: Cattle Fever Tick Eradication Program
SCFT: Southern cattle fever tick
WTD: White-tailed deer
