# Supplementary material for: Effects of white-tailed deer habitat use preferences on southern cattle fever tick eradication: simulating impact on “pasture vacation” strategies
Source: Parasit Vectors. 2021 Feb 8;14:102. doi: 10.1186/s13071-021-04590-z (PMC7869250; doi:10.1186/s13071-021-04590-z)
Supplement: Supplementary file 1 — Additional file 1. Summary of model parameters and equations. [file 13071_2021_4590_MOESM1_ESM.pdf]

Additional file 1

Summary of model parameters and equations

Parameters and equations are based on information presented in Mount et al. [1] unless otherwise specified.

Fecundity (*F*; eggs/engorged female)

Depend on the mean temperature (T; 12℃-32℃) during preoviposition

$F = -7487.5 + 790 \cdot T - 14.5 \cdot T^2$; for ticks obtaining blood meal from cattle

$F = (-565.56 + 59.67 \cdot T - 1.10 \cdot T^2) \times 0.8227$; for ticks obtaining blood meal from white-tailed deer [2]

Eggs

Base survival rates (S_E, under optimal weather conditions) in three different types of pasture

CDW (cumulative degree-weeks) >=36

DT (minimum developmental threshold temperature) = 15

1-4 weeks old, $S_{E1} = \begin{cases} 0.964264\ (improved\ dense\ pasture) \\ 0.9362846\ (unimproved\ light\ pasture) \\ 0.888922\ (improved\ light) \end{cases}$

> 4 weeks old, $S_{E2} = \begin{cases} 0.8344503\ (improved\ dense\ pasture) \\ 0.8015637\ (unimproved\ light\ pasture) \\ 0.7148943\ (improved\ light) \end{cases}$

Note that for habitat-specific tick survival parameters, we have retained the original habitat names in Mount et al. [1]: "improved dense pasture," "unimproved light pasture," and "improved light," which they used to refer to good, fair, and poor habitats, respectively, relative to survival of off-host ticks. These parameter values were based on maximum survival times reported in eight independent studies conducted in a variety of habitats (see page 225 in Mount et al. [1]). In the text describing our study, we have used the names "mesquite savanna," "mixed-brush savanna," and "open un-canopied grassland" to refer to good, fair, and poor habitats, respectively, relative to survival of off-host ticks.

Survival rate effects (SRE) for the temperature (T), saturation deficit (SD), and precipitation index (PI) (the following equation, SRE_{E1} will be used for 1-4 weeks old eggs, 1-6 weeks old larvae, and 1-4 weeks engorged females, and SRE_{old} will be used for > 4 weeks old eggs, > 6 weeks old larvae, and > 4 weeks engorged female as well)

1-4 weeks old, $SRE_{young} = (-0.00097222 \cdot T^2 + 0.03111111 \cdot T + 0.75111111) \times (-0.00123643 \cdot SD^2 + 0.01456543 \cdot SD + 0.95708835) \times (-0.00060557 \cdot PI^2 + 0.0159513 \cdot PI + 0.89479707)$

> 4 weeks old, $SRE_{old} = (-0.00114583 \cdot T^2 + 0.036250 \cdot T + 0.71333333) \times (-0.00141020 \cdot SD^2 + 0.01827455 \cdot SD + 0.94021589) \times (-0.00074267 \cdot PI^2 + 0.0194957 \cdot PI + 0.87183511)$

Free-living (host seeking) larvae

Base survival rates (S_{FL}, under optimal weather conditions) in three different types of pasture

1-6 weeks old, $S_{FL1} = \begin{cases} 0.9539088\ (improved\ dense\ pasture) \\ 0.9342157\ (unimproved\ light\ pasture) \\ 0.9001101\ (improved\ light) \end{cases}$

> 6 weeks old, $S_{FL2} = \begin{cases} 0.9154279\ (improved\ dense\ pasture) \\ 0.8982628\ (unimproved\ light\ pasture) \\ 0.8516867\ (improved\ light) \end{cases}$

Survival rate effects (SRE) for the temperature (T), saturation deficit (SD), and precipitation index (PI)

1-6 weeks old, $SRE_{young} = (-0.00097222 \cdot T^2 + 0.03111111 \cdot T + 0.75111111) \times (-0.00123643 \cdot SD^2 + 0.01456543 \cdot SD + 0.95708835) \times (-0.00060557 \cdot PI^2 + 0.0159513 \cdot PI + 0.89479707)$

> 6 weeks old, $SRE_{old} = (-0.00114583 \cdot T^2 + 0.036250 \cdot T + 0.71333333) \times (-0.00141020 \cdot SD^2 + 0.01827455 \cdot SD + 0.94021589) \times (-0.00074267 \cdot PI^2 + 0.0194957 \cdot PI + 0.87183511)$

Host-finding rate

Relative rates of host-seeking activity of off-host larvae (HFR, the proportion of off-host larvae that potentially could encounter and attach to a host)

$HFR = -0.008 \times T^2 + 0.4 \times T - 4$

On host larvae (density-dependent survival on cattle)

Survival rate (S_L); depends on cattle type and tick density (ticks per host, D)

When tick density (tick exposure index) < 1000, $S_L = 0.37$;

When 1000 <= tick density (tick exposure index) < 15000, $S_L = -0.000013 \cdot D + 0.382857$;

When 15000 <= tick density (tick exposure index), $S_L = 0.19$

On host nymphs

Survival rate (S_N); depends on cattle type and tick density (ticks per host, D)

When tick density (tick exposure index) < 1000, $S_N = 0.97$;

When 1000 <= tick density (tick exposure index) < 15000, $S_N = -0.000027 \cdot D + 0.997143$;

When 15000 <= tick density (tick exposure index), $S_N = 0.59$

On host adults

Survival rate (S_F and S_M); depends on cattle type and tick density (ticks per host, D)

Male and female ticks are separated during the transition of nymphs to adults on host using a constant female/male ratio of 1.36:1.

Male ticks are contained in one accumulator with no age structure.

The time on host is 1 or 2 weeks for females (50% split).

When tick density (tick exposure index) < 1000, $S_F = S_M = 0.79$;

When 1000 <= tick density (tick exposure index) < 15000, $S_F = S_M = -0.000023 \cdot D + 0.812857$;
When 15000 <= tick density (tick exposure index), $S_F = S_M = 0.47$

Engorged adult females

Base survival rates (S_{EF}, under optimal weather conditions) in three different types of pasture

CDW (cumulative degree-weeks) >= 30

DT (minimum developmental threshold temperature) = 15

1-4 weeks old, $S_{EF1} = \begin{cases} 0.974004\ (improved\ dense\ pasture) \\ 0.945742\ (unimproved\ light\ pasture) \\ 0.897901\ (improved\ light) \end{cases}$

> 4 weeks old, $S_{EF2} = \begin{cases} 0.8428791\ (improved\ dense\ pasture) \\ 0.8096603\ (unimproved\ light\ pasture) \\ 0.7221155\ (improved\ light) \end{cases}$

Survival rate effects (SRE) for the temperature (T), saturation deficit (SD), and precipitation index (PI)

1-4 weeks old, $SRE_{young} = (-0.00097222 \cdot T^2 + 0.03111111 \cdot T + 0.75111111) \times (-0.00123643 \cdot SD^2 + 0.01456543 \cdot SD + 0.95708835) \times (-0.00060557 \cdot PI^2 + 0.0159513 \cdot PI + 0.89479707)$

> 4 weeks old, $SRE_{old} = (-0.00114583 \cdot T^2 + 0.036250 \cdot T + 0.71333333) \times (-0.00141020 \cdot SD^2 + 0.01827455 \cdot SD + 0.94021589) \times (-0.00074267 \cdot PI^2 + 0.0194957 \cdot PI + 0.87183511)$

References

1. Mount GA, Haile DG, Davey RB, Cooksey LM. Computer simulation of *Boophilus* cattle tick (Acari: Ixodidae) population dynamics. Journal of Medical Entomology. 1991;28 2:223-40; doi: 10.1093/jmedent/28.2.223. https://doi.org/10.1093/jmedent/28.2.223.
2. Cooksey LM, Davey RB, Ahrens EH, George JE. Suitability of white-tailed deer as hosts for cattle fever ticks (Acari: Ixodidae). Journal of Medical Entomology. 1989;26 3:155-8.
